# Is Almond Consumption More Effective Than Reduced Dietary Saturated Fat at Decreasing Plasma Total Cholesterol and LDL-c Levels? A Theoretical Approach

**DOI:** 10.1155/2012/265712

**Published:** 2012-11-29

**Authors:** Rudy M. Ortiz, Steven Garcia, Arnold D. Kim

**Affiliations:** ^1^Department of Molecular and Cellular Biology, School of Natural Sciences, University of California, Merced, Merced, CA 95343, USA; ^2^Department of Applied Mathematics, University of California, Merced, Merced, CA 95343, USA

## Abstract

Hypercholesterolemia can be a consequence of excessive dietary saturated fatty acid (SFA), while almond-supplemented diets can improve lipid profiles. However, the differential and independent impacts of dietary SFA and almondsupplemented diets on plasma total cholesterol (pTC) and low-density lipoprotein (pLDL-c) concentrations have not been directly compared and are not well described. We reviewed the available data to construct multiple regression analyses to theoretically assess the impact of relative almond intake (RAI) and dietary SFA on reducing pTC and pLDL-c concentrations. Strong, negative correlations between RAI and percent change in mean pTC (*R* = 0.776; *P* = 0.005) and RAI and percent change in mean pLDL-c (*R* = 0.818; *P* = 0.002) were detected. The relationships between percent change in mean dietary SFA, and percent change in mean pTC and mean pLDL-c were weaker and only significant for pLDL-c. The multiple regression analyses demonstrated modest improvements in the strength of the correlations for both pTC (*R* = 0.804; *P* = 0.016) and pLDL-c (*R* = 0.855; *P* = 0.005). The models suggest that the increase in RAI contributes to the reduction in pTC and pLDL-c to a greater extent than a reduction in dietary SFA, but a simultaneous decrease in dietary SFA should further improve lipid profiles.

## 1. Introduction

Chronic hypercholesterolemia is a critical risk factor in the development of cardiovascular disease (CVD), which is the most common cause of death worldwide [1–4]. Furthermore, low-density lipoprotein (LDL) is the major atherogenic lipoprotein and the primary target of cholesterol-lowering therapy because numerous clinical trials have demonstrated the efficacy of LDL-lowering therapy for reducing the risk of CVD [3, 4]. A recognized consequence of increased dietary saturated fatty acid (SFA) is hypercholesterolemia [5–11]. Conversely, diets supplemented with almonds or almond products (i.e., oil and butter) have been shown to produce a moderate, yet significant decrease in plasma total cholesterol (pTC) (3–11%) and plasma LDL cholesterol (pLDL-c) (3–18%) [12–23], which demonstrates a potential benefit from consuming almonds on improving cardiovascular health. For these reasons, studies of natural foods that have the potential to significantly improve circulating lipid profiles, especially reducing pLDL-c, are of particular importance. The nutriceutical benefits of nuts provide promise for taking a dietary approach to addressing the increasing prevalence of CVD globally. The mechanisms by which nuts and nut-supplemented diets contribute to reduced pTC and pLDL-c have not been revealed, and, given the nature of these types of studies, elucidation of these mechanisms in humans is not likely. Thus, the intriguing question of how nuts induce a cholesterol-lowering benefit remains. Is the effect simply and strictly displacement or do nuts reduce *de novo* cholesterol synthesis? In the interim, theoretical studies that provide a better understanding of the effects of almond-supplemented diets on plasma cholesterol will serve a meaningful purpose to this end and provide further insight on the impacts of dietary interactions among different foods. 

A modeling approach to better understand the impacts of dietary fats and nut consumption on plasma cholesterol has been realized [24]. This highly innovative approach and significant contribution to the area of nut consumption and circulating lipids examined the effects of substituting saturated fat intake with monounsaturated and polyunsaturated fatty acids by varying the consumption of various nuts [24]. This study acknowledged that the levels of dietary SFA may be manipulated by the consumption of nuts, which is an effective strategy for reducing pLDL-c concentrations, and for preventing a reduction in HDL-cholesterol and an increase in plasma triglyceride induced by low fat, high carbohydrate diets [24]. However, this comprehensive and elegant meta-analysis examined all nuts and did not specifically focus on assessing covariate effects of dietary SFA and almond supplementation on changes in plasma cholesterol. Furthermore, we took an alternative approach to assessing almond consumption by examining consumption as a function of body mass. Most studies report nut consumption as a fixed variable without consideration for a potential effect of changes in body mass, which is a tenet of pharmacological studies. That is, we wanted to evaluate if a dose-dependent effect of almond consumption on plasma cholesterol (TC and LDL-c) existed, which has not been presented previously. Thus, the current study was conceived in a manner to complement the significant contributions of those previously described [24]. Therefore, we modeled the more recent data on the effects of almond-supplemented diets on plasma cholesterol to address the hypothesis that relative almond intake has a greater impact on reducing plasma cholesterol than dietary SFA.

## 2. Methods

A PubMed search for peer-reviewed publications on the effects of almonds and almond-supplemented diets on plasma cholesterol was conducted. Twenty-one studies were found that reported on the effects of diet and almond supplementation on pTC, and pLDL-c levels. While each study implemented different diets, the present analyses were based on reported mean values for the amount of almonds consumed, body mass (BM), dietary SFA, pTC and pLDL-c. Thus, these inclusion criteria had to be reported in a manner that percent changes in mean dietary SFA, pTC, pLDL-c, and relative almond consumption (RAI; almond intake as a function of body mass) between initial and final measurement periods could be calculated [12, 18–22] with one exception [15].

### 2.1. Relative Almond Intake

Because the amount of almond supplementation within a particular study is fixed, despite differences in BM of participants, almond consumption across studies was normalized to account for these differences in mean BM. Thus, RAI was calculated and presented as g/kg BM. In theory, this approach should help alleviate the impact of the differences in mean BM among the study subjects and also help normalize for differences in almond doses used among the different studies. Because Hyson et al. [15] reported BMI and not BM, BM was derived from mean BMI assuming an average height for their study population of 1.7526 m (69 in) given that more women (*n* = 17) than men (*n* = 14) comprised their study population. Furthermore, we calculated RAI using a range of assumed heights (69 ± 3 in) and the, at most, 8% difference in RAI (0.84 g/kg BM) did not significantly alter the regression values.

### 2.2. Change in Dietary Saturated Fatty Acids

The change in mean dietary SFA was calculated as percent change from baseline to account for the differences in how values were reported (i.e., percentage of energy or g/d).

### 2.3. Changes in Plasma Total Cholesterol and Low-Density Lipoprotein

Similarly, because pTC and pLDL-c were reported in both standard (mg/dL) and metric (mM) units among the different studies, this difference was accounted for by calculating percent change in pTC and pLDL-c between initial and final measurement periods in each study. 

### 2.4. Statistical Analyses

Relative almond consumption was identified as a contributing factor to a reduction in pTC and pLDL-c. The effect of dietary SFA intake on pLDL-c was moderately strong (*R* = 0.624), but significant (*P* = 0.040) and the effect on pTC was similarly as strong (*R* = 0.567), but only borderline nonsignificant (*P* = 0.069). Thus, each factor alone was not able to sufficiently account for the changes in plasma TC or LDL-c. Therefore, a multivariate regression model was used where *X*_1_ denoted relative almond intake, *X*_2_ denoted the percent change in dietary SFA, and *Y* denoted the percent change in plasma TC or LDL-c. The multivariate, linear regression model used to fit the data was defined by

(1) $$\begin{array}{c} Y=\beta_{0}+\beta_{1}X_{1}+\beta_{2}X_{2}. \end{array}$$

The coefficients *β*_0_, *β*_1_, and *β*_2_ were computed using ordinary least squares applied to the data collected. The coefficient of determination or *R* ^2^ value was computed to assess the validity of this multivariate, linear regression model. Scatter plots of the data provided a method to qualitatively compare the model to the data. Moreover, 2 data points (control group from Spiller et al. [22] and raw-almond group from Tovar et al. [9]) were identified as statistical outliers and not considered in the regression models here. The slopes of the regression analyses were compared by analysis of covariance (ANCOVA). Regressions and slopes were considered significant at *P* < 0.05. Models were constructed and means compared using STAT software (version 3.0; Richmond, VT).

## 3. Results

### 3.1. Relative Almond Intake and Plasma Total Cholesterol and Low-Density Lipoprotein

**Figure 1:**
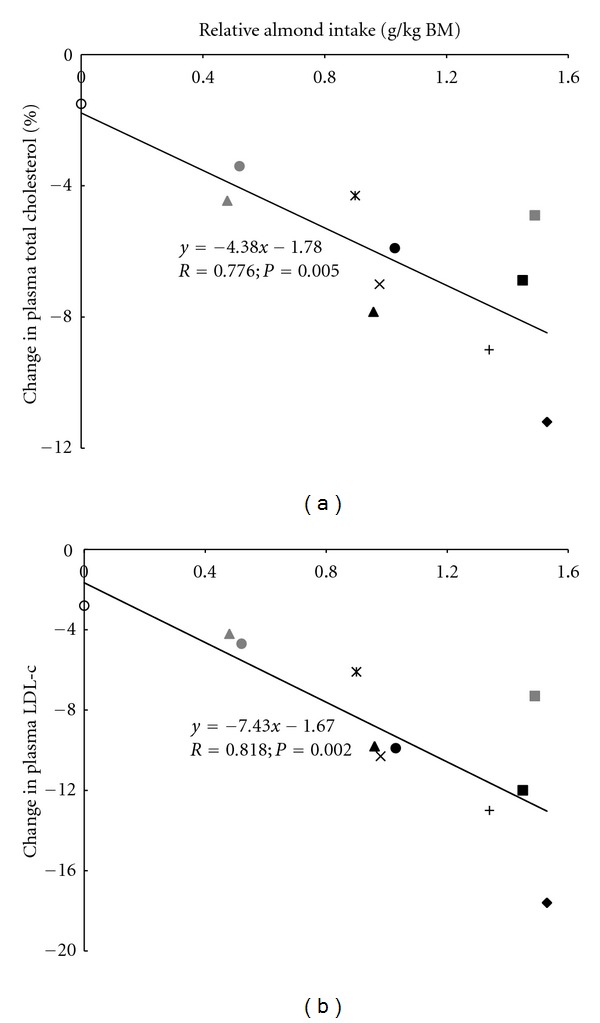
Correlation between mean relative almond intake (RAI) and (a) percent change in mean plasma total cholesterol (pTC) and (b) percent change in mean plasma low-density lipoprotein cholesterol (pLDL-c). Correlations were considered significant at *P* < 0.05. Open circle: Jenkins et al. [18] (control diet); plus sign: Spiller et al. [20]; “X”: Abbey et al. [12]; diamond: Spiller et al. [22]; cross-thru “X”: Hyson et al. [15]; gray triangle: Sabaté et al. [19] (low-almond diet); black triangle: Sabaté et al. [19] [high-almond diet]; gray square: Spiller et al. [21] (roasted-almond diet); black square: Spiller et al. [21] (raw-almond diet); gray circle: Jenkins et al. [18] [half-almond diet]; black circle: Jenkins et al. [18] (full-almond diet).

The compiled analyses demonstrated a strong (*R* = 0.776), negative, and significant (*P* = 0.005) relationship between percent change in mean RAI and percent change in mean pTC (Figure 1(a)). The relationship between percent change in mean RAI and percent change in mean pLDL-c was slightly stronger (*R* = 0.818), but more significant (*P* = 0.002) than that for percent change in mean pTC (Figure 1(b)). The slopes were not different (*P* > 0.10) between the two relationships.

### 3.2. Dietary SFA Intake and Plasma Cholesterol and Low-Density Lipoprotein

While a moderately strong, positive (*y* = 0.11*x* − 3.74; *R* = 0.567) relationship between percent change in dietary SFA intake and percent change in mean pTC was detected, this relationship was not statistically significant (*P* = 0.069). As with the relationship between RAI and pLDL-c, the relationship between percent change in dietary SFA intake and percent change in mean pLDL-c (*y* = 0.19*x* − 4.82) was stronger (*R* = 0.624) than that for pTC and was significant (*P* = 0.040). The slopes were not different (*P* > 0.10) between the two relationships.

### 3.3. Multiple Regression Analyses

**Figure 2:**
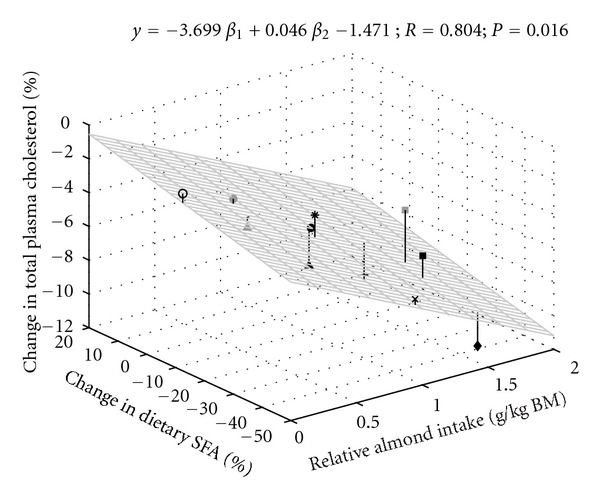
Multiple regression analysis of independent variables (mean relative almond intake and percent change in mean dietary saturated fatty acid) and percent change in mean plasma total cholesterol. Correlation was considered significant at *P* < 0.05. See Figure 1 legend for symbol identification.

**Figure 3:**
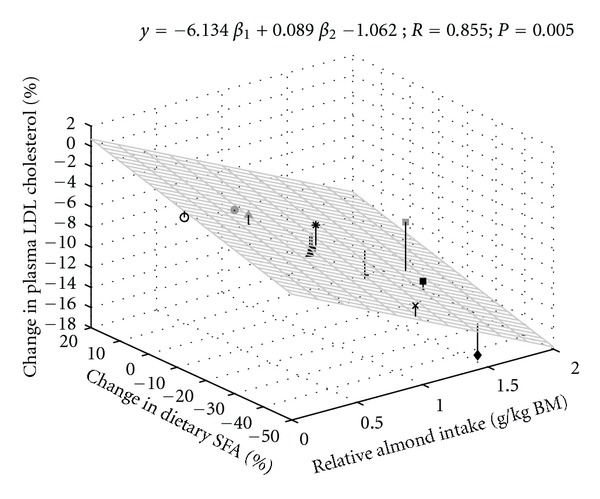
Multiple regression analysis of independent variables (mean relative almond intake and percent change in mean dietary saturated fatty acid) and percent change in mean plasma low-density lipoprotein cholesterol. Correlation was considered significant at *P* < 0.05. See Figure 1 legend for symbol identification.

To more thoroughly evaluate the effects of multiple factors known to impact pTC and pLDL-c, multiple regression analysis was performed. These analyses demonstrated strong and significant relationships between the independent variables percent change in mean RAI and percent change in mean dietary SFA intake) and percent change in mean pTC (Figure 2) or percent change in mean pLDL-c (Figure 3), with the effects greater on pLDL-c than on pTC.

## 4. Discussion

Hypercholesterolemia continues to serve as the most predictive risk factor for the development of cardiovascular disease (CVD) [1–4, 25], and because LDL-c constitutes a majority of total circulating cholesterol, pLDL-c is the primary target for cholesterol-lowering therapies [3, 4, 25]. In addition to genetic factors that contribute to hypercholesterolemia [26], excessive consumption of dietary SFA may also contribute to increased plasma cholesterol (total and LDL) levels [11]. A variety of nuts (almonds, walnuts, pistachios, peanuts, and macadamia nuts) have been reported to possess cholesterol-lowering benefits [5, 6, 8–10, 13–24, 27–34] suggesting that dietary modifications have the potential to improve lipid profiles and ultimately abate the prevalence of CVD. Therefore, a more thorough analysis of the theoretical relationships among dietary SFA, almond consumption, and plasma cholesterol could prove worthwhile in assessing the potential benefits of dietary modifications. 

The most significant finding of the present analyses suggests that increased almond consumption has theoretically a greater potential to reduce both plasma total and LDL cholesterol than reduced dietary saturated fatty acids alone. This is corroborated by the fact that strong and highly significant relationships between RAI and pTC and pLDL-c were detected. Conversely, the relationships between percent change in mean dietary SFA and pTC and pLDL-c were only moderately strong, and only significant for pLDL-c. Along these lines, the impact of increased RAI was greater on reducing percent change in mean pLDL-c than on mean pTC suggesting that the benefits of increased almond consumption on lowering cholesterol could be therapeutic because of their effectiveness on the primary target (LDL-c) of pharmacological interventions. While it is likely that an increase in sample size will lead to a significant relationship between percent change in dietary SFA and pTC, the strength of the relationships between RAI and both pTC and pLDL-c were consistently stronger than those with dietary SFA suggesting that RAI has a greater effect on pTC and pLDL-c than reduced dietary SFA intake. The multiple regression analyses demonstrate that the inclusion of the dietary SFA data in the analyses only modestly improves the strength of the regressions for pTC (+3.6%) and pLDL-c (+4.5%) further suggesting that increased RAI is the primary contributor to the reductions in mean pTC and pLDL-c. Nonetheless, reducing dietary SFA in addition to increasing almond consumption has a greater potential for reducing plasma cholesterol (total and LDL) than either has alone. Alternatively, the results also suggest that increased or the lack of a reduction in dietary SFA intake may impair the ability of almonds to reduce plasma cholesterol levels. Thus, to realize the greatest benefit of the consumption of almonds, and possibly other nuts, on plasma total and LDL cholesterol, a simultaneous reduction in dietary SFA intake would be recommended.

**Limitations**

As is the case with most theoretical studies, we are cognizant of limitations in the approach and interpretations. Because most of the studies we reviewed targeted reducing pTC and pLDL-c as principal outcomes, it should not be completely unexpected that the impact of increased RAI was greater than reduced dietary SFA on pTC and pLDL-c. Also, comparing the effects of both independent variables is complicated because studies reviewed here could not completely control for changes in dietary SFA as some studies tried to displace dietary SFA with almond fats. Future studies where dietary SFA and almond fats can be better controlled will help alleviate this limitation. But this limitation is more likely a factor of study participant compliance than actual study design, so this issue may linger in future studies. The inclusion of only 7 out of 21 papers identified also limits the impact of these findings. Additionally, because so few studies exist on the effects of other nuts on lipid profiles such theoretical analyses are limited to almonds. However, comparisons among different nuts would be interesting to determine if nuts as a group are equivalent in terms of their effects on blood lipid profiles. Nonetheless, given these limitations, the fact that significant relationships could be detected suggests that almonds, and possibly other nuts, have a practical effect on improving plasma cholesterol, largely independent of reductions in dietary SFA.

## 5. Conclusions

The ramification of the calculated relationships is that the benefits of almond consumption on reducing plasma total and LDL cholesterol are greater than reduced dietary SFA intake alone. While we recognize and demonstrate the importance reduced dietary SFA has on reducing plasma total and LDL cholesterol, the present estimations suggest that increased almond intake is more beneficial. Furthermore, the nature of the relationships would suggest that the cholesterol-lowering effects of almonds are, at least in part, a function of displacement. Future animal studies designed to specifically address the effects of almonds on displacement versus *de novo* synthesis would be impactful and highly informative. Nonetheless, if the relationships are truly linear, then the greater the reduction in dietary SFA intake, the more impactful the cholesterol-lowering effects of almonds should be. How therapeutic these benefits are at ameliorating CVD requires further investigation, but these analyses provide legitimacy for the pursuit of such studies. Moreover, it would be interesting to learn if the benefits of RAI on pTC and pLDL-c are saturable so that an upper-limit of almond intake can be identified with respect to optimizing the benefit on reducing plasma cholesterol. Furthermore, a study that simultaneously controls for both relative almond consumption and dietary SFA intake would provide further insight on the contribution of each variable to reducing plasma cholesterol. Whether or not a similar relationship can be established for other nuts or foods would be interesting and could be beneficial when producing dietary recommendations for those particular foods, especially when considering dietary SFA.

## References

[1] http://www.heart.org/HEARTORG/Conditions/Cholesterol/AboutCholesterol/About-Cholesterol_UCM_001220_Article.jsp#.Txcyh2-JcQo.

[2] Bitton A, Gaziano T (2010). The Framingham Heart Study’s impact on global risk assessment. *Progress in Cardiovascular Diseases*.

[3] Mihaylova B, Emberson J, Blackwell L (2012). The effects of lowering LDL cholesterol with statin therapy in people at low risk of vascular disease: meta-analysis of individual data from 27 randomised trials. *The Lancet*.

[4] (2002). Third report of the National Cholesterol Education Program (NCEP) expert panel on detection, evaluation, and treatment of high blood cholesterol in adults (Adult Treatment Panel III) final report. *Circulation*.

[5] Han SN, Leka LS, Lichtenstein AH, Ausman LM, Schaefer EJ, Meydani SN (2002). Effect of hydrogenated and saturated, relative to polyunsaturated, fat on immune and inflammatory responses of adults with moderate hypercholesterolemia. *Journal of Lipid Research*.

[6] Katan MB (2006). Alternatives to low-fat diets. *American Journal of Clinical Nutrition*.

[7] Lefevre M, Champagne CM, Tulley RT, Rood JC, Most MM (2005). Individual variability in cardiovascular disease risk factor responses to low-fat and low-saturated-fat diets in men: body mass index, adiposity, and insulin resistance predict changes in LDL cholesterol. *American Journal of Clinical Nutrition*.

[8] Schaefer EJ (2002). Lipoproteins, nutrition, and heart disease. *American Journal of Clinical Nutrition*.

[9] Tovar J, Nilsson A, Johansson M (2012). A diet based on multiple functional concepts improves cardiometabolic risk parameters in healthy subjects. *Nutrition and Metabolism*.

[10] Trautwein EA, Rieckhoff D, Kunath-Rau A, Erbersdobler HF (1999). Replacing saturated fat with PUFA-rich (sunflower oil) or MUFA-rich (rapeseed, olive and high-oleic sunflower oil) fats resulted in comparable hypocholesterolemic effects in cholesterol-fed hamsters. *Annals of Nutrition and Metabolism*.

[11] Zafra MF, Castillo M, Rodriguez-Vico F, Garcia-Peregrin E (1992). Induction in *Gallus domesticus* of experimental hypercholesterolemia by saturated fat. Effects on cholesterogenic enzyme activity. *Archives Internationales de Physiologie, de Biochimie et de Biophysique*.

[12] Abbey M, Noakes M, Belling GB, Nestel PJ (1994). Partial replacement of saturated fatty acids with almonds or walnuts lowers total plasma cholesterol and low-density-lipoprotein cholesterol. *American Journal of Clinical Nutrition*.

[13] Fraser GE (1999). Nut consumption, lipids, and risk of a coronary event. *Clinical Cardiology*.

[14] Hu FB, Stampfer MJ (1999). Nut consumption and risk of coronary heart disease: a review of epidemiologic evidence. *Current Atherosclerosis Reports*.

[15] Hyson DA, Schneeman BO, Davis PA (2002). Almonds and almond oil have similar effects on plasma lipids and LDL oxidation in healthy men and women. *Journal of Nutrition*.

[16] Jenkins DJA, Kendall CWC, Marchie A (2003). The effect of combining plant sterols, soy protein, viscous fibers, and almonds in treating hypercholesterolemia. *Metabolism*.

[17] Jenkins DJA, Kendall CWC, Marchie A (2003). The Garden of Eden—plant based diets, the genetic drive to conserve cholesterol and its implications for heart disease in the 21st century. *Comparative Biochemistry and Physiology A*.

[18] Jenkins DJA, Kendall CWC, Marchie A (2008). Almonds reduce biomarkers of lipid peroxidation in older hyperlipidemic subjects. *Journal of Nutrition*.

[19] Sabaté J, Haddad E, Tanzman JS, Jambazian P, Rajaram S (2003). Serum lipid response to the graduated enrichment of a Step I diet with almonds: a randomized feeding trial. *American Journal of Clinical Nutrition*.

[20] Spiller GA, Jenkins DJA, Cragen LN (1992). Effect of a diet high in monounsaturated fat from almonds on plasma cholesterol and lipoproteins. *Journal of the American College of Nutrition*.

[21] Spiller GA, Miller A, Olivera K (2003). Effects of plant-based diets high in raw or roasted almonds, or roasted almond butter on serum lipoproteins in humans. *Journal of the American College of Nutrition*.

[22] Spiller GA, Jenkins DAJ, Bosello O, Gates JE, Cragen LN, Bruce B (1998). Nuts and plasma lipids: an almond-based diet lowers LDL-C while preserving HDL-C. *Journal of the American College of Nutrition*.

[23] Wien MA, Sabaté JM, Iklé DN, Cole SE, Kandeel FR (2003). Almonds vs complex carbohydrates in a weight reduction program. *International Journal of Obesity*.

[24] Kris-Etherton PM, Yu-Poth S, Sabaté J, Ratcliffe HE, Zhao G, Etherton TD (1999). Nuts and their bioactive constituents: effects on serum lipids and other factors that affect disease risk. *American Journal of Clinical Nutrition*.

[25] Skilton MR, Moulin P, Sérusclat A, Nony P, Bonnet F (2007). A comparison of the NCEP-ATPIII, IDF and AHA/NHLBI metabolic syndrome definitions with relation to early carotid atherosclerosis in subjects with hypercholesterolemia or at risk of CVD: evidence for sex-specific differences. *Atherosclerosis*.

[26] Slimani A, Jelassi A, Jguirim I (2012). Effect of mutations in LDLR and PCSK9 genes on phenotypic variablilty in Tunisian familial hypercholesterolemia patients. *Atheroscler*.

[27] Cox C, Mann J, Sutherland W, Chisholm A, Skeaff M (1995). Effects of coconut oil, butter, and safflower oil on lipids and lipoproteins in persons with moderately elevated cholesterol levels. *Journal of Lipid Research*.

[28] Alper CM, Mattes RD (2003). Peanut consumption improves indices of cardiovascular disease risk in healthy adults. *Journal of the American College of Nutrition*.

[29] Gebauer SK, West SG, Kay CD, Alaupovic P, Bagshaw D, Kris-Etherton PM (2008). Effects of pistachios on cardiovascular disease risk factors and potential mechanisms of action: a dose-response study. *American Journal of Clinical Nutrition*.

[30] Griel AE, Cao Y, Bagshaw DD, Cifelli AM, Holub B, Kris-Etherton PM (2008). A Macadamia nut-rich diet reduces total and LDL-cholesterol in mildly hypercholesterolemic men and women. *Journal of Nutrition*.

[31] Hollis J, Mattes R (2007). Effect of chronic consumption of almonds on body weight in healthy humans. *British Journal of Nutrition*.

[32] Li TY, Brennan AM, Wedick NM, Mantzoros C, Rifai N, Hu FB (2009). Regular consumption of nuts is associated with a lower risk of cardiovascular disease in women with type 2 diabetes. *Journal of Nutrition*.

[33] Olmedilla-Alonso B, Granado-Lorencio F, Herrero-Barbudo C, Blanco-Navarro I, Blázquez-García S, Pérez-Sacristán B (2008). Consumption of restructured meat products with added walnuts has a cholesterol-lowering effect in subjects at high cardiovascular risk: a randomised, crossover, placebo-controlled study. *Journal of the American College of Nutrition*.

[34] Tey SL, Brown R, Gray A, Chisholm A, Delahunty C (2011). Nuts improve diet quality compared to other energy-dense snacks while maintaining body weight. *Journal of Nutrition and Metabolism*.

